# Natural Language Processing as an Emerging Tool to Detect Late-Life Depression

**DOI:** 10.3389/fpsyt.2021.719125

**Published:** 2021-09-06

**Authors:** Danielle D. DeSouza, Jessica Robin, Melisa Gumus, Anthony Yeung

**Affiliations:** ^1^Winterlight Labs, Toronto, ON, Canada; ^2^Department of Psychiatry, University of Toronto, Toronto, ON, Canada

**Keywords:** geriatric mental health, depression, speech, natural language processing, artificial intelligence, digital health, late-life depression

## Abstract

Late-life depression (LLD) is a major public health concern. Despite the availability of effective treatments for depression, barriers to screening and diagnosis still exist. The use of current standardized depression assessments can lead to underdiagnosis or misdiagnosis due to subjective symptom reporting and the distinct cognitive, psychomotor, and somatic features of LLD. To overcome these limitations, there has been a growing interest in the development of objective measures of depression using artificial intelligence (AI) technologies such as natural language processing (NLP). NLP approaches focus on the analysis of acoustic and linguistic aspects of human language derived from text and speech and can be integrated with machine learning approaches to classify depression and its severity. In this review, we will provide rationale for the use of NLP methods to study depression using speech, summarize previous research using NLP in LLD, compare findings to younger adults with depression and older adults with other clinical conditions, and discuss future directions including the use of complementary AI strategies to fully capture the spectrum of LLD.

## Introduction

Depression is one of the leading causes of disability worldwide, affecting more than 264 million people of all ages (1). Although less prevalent among older adults (2), late-life depression (LLD), also referred to as geriatric depression, remains a major public health concern due to increased risk of morbidity, suicide, physical, cognitive, and social impairments, and self-neglect (3, 4). With a progressively aging population globally, the identification and treatment of LLD is critical (5).

LLD is generally defined as depression occurring in individuals aged 60 and over, though cutoffs vary in the literature. LLD can be further divided into early onset (first depressive episode before age 60) and late onset (first depressive episode after age 60) (6). For the purposes of this review, the definition of LLD includes both early and late onset episodes (5, 7, 8). As with younger individuals, LLD can be heterogeneous ranging from subthreshold changes in mood to major depression as outlined by the Diagnostic and Statistical Manual of Mental Disorders (DSM-5). However, diagnosing LLD is more challenging due to a different symptom profile compared to younger adults, and additional medical comorbidities (5, 9, 10). Misdiagnosis can occur if classic depressive symptoms (e.g., low mood) are not verbally expressed, and instead only somatic or cognitive symptoms are reported (11). Current depression treatment guidelines also recommend the use of standardized rating scales to gauge symptom severity (5), however, these scales may over or under emphasize the presence of somatic symptoms. One recent review of LLD scales suggested that the over-reliance on somatic items may result in a misdiagnosis of LLD due to the high prevalence of medical comorbidities in older adults (12). Additionally only a handful of assessments, including the Patient Health Questionnaire-9 (PHQ-9), Cornell Scale for Depression in Dementia, Geriatric Depression Scale, and the Hospital Anxiety and Depression Scale have specifically been validated in LLD (13–16). However, these validated scales can be susceptible to bias due to the subjective nature of scoring by the assessing clinician. These scales might also falsely identify individuals with cognitive impairment as depressed (10, 17).

To help overcome these limitations, there has been a growing interest in the development of objective measures of depression using artificial intelligence (AI) technologies such as natural language processing (NLP) (18–20). NLP approaches focus on the analysis of acoustic and linguistic aspects of human language derived from speech and text and can be integrated with machine learning approaches to classify depression and its severity (19, 21). Advantages of using these approaches to understand depression symptoms through speech include high ecological validity, low subjectivity, low cost of frequent assessments, and quicker administration of tasks compared to standard assessments. An added benefit of speech analysis using NLP is that speech data can be collected remotely, meeting a vital need for remote cognitive and behavioral assessments in the era of the coronavirus disease (COVID-19) pandemic (22). In this review we will provide rationale for the use of NLP approaches to study depression using speech, summarize previous research using NLP in LLD, compare findings to younger adults with depression and older adults with other clinical conditions, and discuss future directions including the use of complementary AI strategies to fully capture the spectrum of LLD symptoms.

To search for relevant literature related to speech analysis in individuals with depression or LLD, PubMed/MEDLINE, Web of Science, and Google Scholar databases were searched using terms including: “geriatric depression”, “older adult depression”, “late-life depression”, “major depressive disorder”, “natural language processing”, “speech analysis”, “speech”, “acoustics”, “linguistics”, “voice”. A sample search query used in the PubMed database is: [(“geriatric depression” OR “late-life depression”) AND (“speech” OR “linguistic” OR “acoustic” OR “language” or “voice”)]. While this mini review was not intended to be a systematic review of all literature related to NLP and depression, we used broad search terms to capture as many studies as possible specifically related to NLP in LLD. Only English language studies were included in the search strategy and no restrictions were placed on the year of publication.

## Understanding Depression Through Speech Analysis

**Figure 1:**
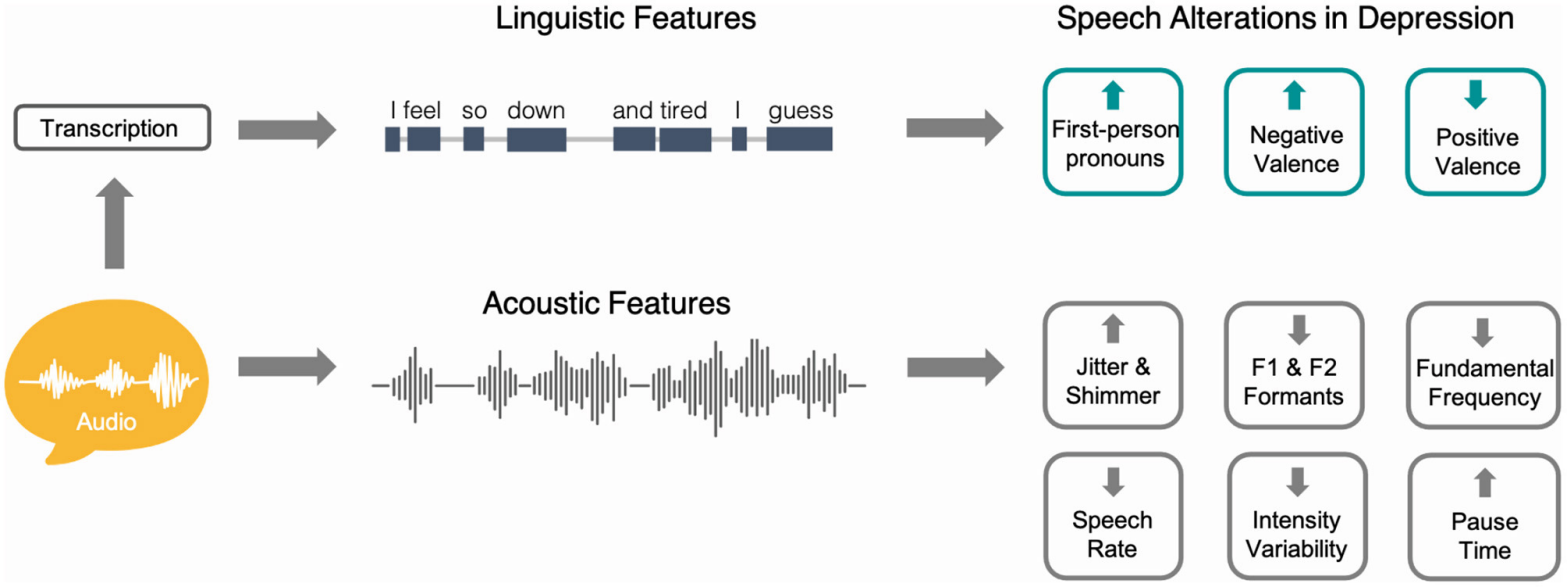
Schematic representation showing how speech can be used to study linguistic and acoustic alterations in depression. To assess linguistic aspects of speech, audio samples are first transcribed using either manual or automatic speech recognition processes. Natural language processing methods can then be used to assess lexical, syntactic, and content measures of language. Audio waveforms can be directly analyzed to capture acoustic aspects of speech such as pause time, speech rate, and fundamental frequency. The right aspect of the figure shows examples of commonly reported speech alterations in the depression literature and the direction of these alterations compared to controls.

Speech production is a complex process involving the communication of thoughts, ideas, and emotions by way of spoken words and phrases. Variations in physiology, cognition, and mood can produce noticeable changes in speech assessed by measures that capture *what* is being said through word selections and grammar usage (linguistic features) and *how* people sound based on acoustic waveforms (acoustic features) (Figure 1). For over a century, clinicians have documented subtle alterations in speech patterns in individuals with depression with early reports highlighting speech that was lower in pitch, more monotonous, slower, and more hesitant (23). These observations were most consistently seen in melancholic and psychotic depression, both of which are characterized by psychomotor retardation (24), a core feature of major depressive disorder (MDD). Other early studies investigating speech in the context of depression and psychomotor retardation reported paucity of speech, lower volume and tone, slowed responses, and monotonous speech (24, 25). Slowed speech or “speaking so slowly that other people could have noticed” is now routinely analyzed as part of self-report depression assessments such as the PHQ-9 (26).

With regard to temporal characteristics of speech in depression, speech pause time or the amount of time between utterances, has been studied since the 1940s. During this period, timing devices could be used to measure speech pause times by manually pressing switches to indicate the start and end of a pause. In one study that included a broad group of individuals with psychiatric diagnoses, it was shown that patients with depression had more silent periods compared to those with hypomania (27). Later research improved study design by more precisely grouping patients according to clinical presentation (e.g., unipolar vs. bipolar, endogenous vs. neurotic) or by using specific tasks to elicit speech. In a small pilot study by Szabadi and colleagues, speech pause time was assessed in individuals with unipolar depression and in healthy controls during a counting task (28). The results showed that participants with depression had elongated speech pause time compared to controls, whose speech pause time remained consistent over a period of 2 months. Importantly, after recovery, pause time alterations normalized in patients, suggesting a role for speech pause time as a marker of clinical improvement. In a series of follow-up studies, Greden et al. replicated the elongated speech pause time findings in larger samples of individuals with depression (29, 30). Hardy and colleagues additionally showed that changes in speech pause time between baseline and final evaluations following a course of treatment for depression were associated with clinical changes on the Retardation Rating Scale for Depression (31). Others have not replicated this finding but have instead suggested that speech pause time abnormalities may be more evident during certain speech tasks (e.g., counting task) and/or only reflect certain depression symptoms or subtypes (32).

Focusing on the linguistic aspects of speech, early studies of depression used psycholinguistic approaches to manually encode measures characterizing lexical diversity, syntactic complexity, and speech content. In one study, comparing individuals with depression to those with mania, depressed participants used more modifying adverbs, first-person pronouns, and personal pronouns. In contrast, participants with mania used more action verbs, adjectives, and concrete nouns (33). Through content analysis, it was also shown that depressed patients used more words indicating self-preoccupation, in line with the large body of literature indicating a role for increased self-rumination in depression [e.g., (34, 35)].

While foundational to our understanding of speech alterations in depression, many early studies relied on traditional approaches such as expert opinion or manual linguistic annotation that have known limitations including subjectivity and limited application to large-scale studies or clinical settings (36). With improved technology, supported by advances in AI, the ability to detect and objectively quantify speech alterations in depression has drastically improved. NLP is a branch of AI that specifically focuses on understanding, interpreting, and manipulating large amounts of human language and speech data. It combines computational linguistics with statistical, machine learning, and deep learning models, to take unstructured, free-form data (e.g., a voice recording or writing sample) and produce structured, quantitative acoustic and linguistic outputs. NLP has the potential to capture the speech changes in depression that reflect both physiologic changes at the basic motor level and also higher-level cognitive processing.

A substantial literature now exists examining automated assessments for depression using speech analysis, with acoustic or paralinguistic speech properties being the focus of multiple in-depth reviews (19, 21). In general, a number of acoustic measures characterized by source features from the vocal folds [e.g., jitter, shimmer, harmonics-to-noise ratio (HNR)], filter features from the vocal tract (e.g., F1 and F2 formants), spectral features [e.g., Mel Frequency Cepstral Coefficients (MFCCs)], and prosodic/melodic features [e.g., fundamental frequency (F0), speech intensity, speed, and pause duration] have been shown to be altered in individuals with depression (19, 21). While some heterogeneity exists in the direction of the reported alterations for some features (e.g., loudness), several trends corroborating earlier studies are evident. For example, greater depression severity is frequently associated with decreased F0 or pitch, intensity variability, and speech rate reflecting slower, more monotonous speech patterns (19, 37–41). Other acoustic measures such as jitter, shimmer, and HNR tend to be higher in depressed patients (19), reflecting laryngeal muscle tension, typically perceived as breathy, rough, or hoarse voices (42). In classification models, MFCCs have been shown to discriminate depressed patients from controls with high sensitivity and specificity (43–45). MFCCs have also been shown to classify the speech of stressed individuals (46). Since depressed language can also be associated with stress (47), future research to disentangle how MFCCs are altered in various cognitive and emotional states is warranted.

Computerized analysis of linguistic speech measures in depression is becoming more common with methods such as Linguistic Inquiry and Word Count (LIWC) (48), which has advantages such as high inter-rater reliability, objectivity, and cost-effectiveness compared to earlier manual approaches. Using LIWC, a recent study showed that participants with depression used more verbal utterances related to sadness compared to individuals with anxiety or comorbid depression and anxiety; however, the groups did not differ in the use of first-person singular pronouns (49). Others have used LIWC to develop composite measures tapping into first-person singular pronoun use, negative affect words, and positive affect words to capture linguistic patterns of depressed affect in nonclinical samples (47). To best capture depression symptom heterogeneity, automated speech analysis methods combining acoustic and linguistic measures may prove to be the most informative (50).

## Speech Patterns in LLD

Given previous work establishing the relationship between speech alterations in depression, researchers have started investigating how speech may be altered in LLD, specifically. Table 1 summarizes recent literature on the topic and highlights different approaches used to collect and analyze speech data. While it is difficult to draw conclusions based on heterogeneous samples and methods, automated speech analysis is proving to be a promising means to tap into cognitive and depressive symptoms in LLD and can be readily adapted for naturalistic settings (55). Encouraging findings indicate that vocal measures can predict high and low depression scores in LLD between 86 and 92% of the time (54). Others have shown that LLD can be classified with 77–86% accuracy compared to age-matched controls (56). In the latter study, acoustic features contributing to accuracy values differed between sexes, highlighting the importance of taking demographic factors such as age, sex, and education into account. Speech has been shown to differ based on these variables even in the absence of clinical conditions. For example, morphological differences in vocal fold length between males and females contribute variation in acoustic features such as F0 (58).

**Table 1:** Summary of recent research examining speech in older adults with depression.

**References**	**Participants**	**Study objective**	**Speech tasks and measures**	**Main findings**
Alpert et al. (39).	22 participants (60+ years, 12 M, 10 F) meeting DSM-III-R depression criteria and 19 age-matched controls (8 M, 11 F).	To measure speech fluency and prosody in elderly depressed patients participating in a treatment trial. Participants were grouped as “agitated” or “retarded” based on clinical ratings.	Counting, reading, and free speech tasks. Acoustic measures tapping into fluency (speech productivity and pausing) and prosody (emphasis and inflection) were analyzed.	Older depressed participants had briefer utterances and less prosodic speech compared to controls. After treatment, improvement in the “retarded” group was associated with briefer pauses.
Murray et al. (51).	18 participants with depression (60–90 years), 17 with Alzheimer's dementia, and 14 age-matched controls.	To determine if depression is associated with changes in discourse patterns and if this discriminates depression from early-stage Alzheimer's disease.	Picture description, sentence reading, and validated memory and language tasks. Quantitative, syntactic, and informativeness aspects of speech were analyzed.	Alzheimer's participants produced more uninformative utterances than depression and controls. No differences in informativeness between depression and controls.
Rabbi et al. (52).	Eight older individuals (4 M, 4 F) from a continuing care retirement community. Depression assessed using the CES-D (53). The SF-36 assessed overall well-being including mental health.	To demonstrate the feasibility of a multi-modal mobile sensing system to simultaneously assess mental and physical health in older individuals.	Authors measured the ratio of time speech was detected relative to the duration of the audio recording.	Amount of detected speech was positively associated with overall well-being.
Smith et al. (54).	46 older adults (66–93 years, 10 M, 36 F) recruited from senior living communities. Depression symptoms assessed using the PHQ-9.	To determine if vocal alterations associated with clinical depression in younger adults are also indicative of depression in older adults.	Reading out loud and free speech and were collected two weeks apart. Speech measures included F0, jitter, shimmer, loudness, MFCCs, and LPCCs.	Speech features predicted high and low depression scores between 86 and 92% of the time. Changes in raw PHQ-9 scores were predicted within 1.17 points.
Little et al. (55).	29 individuals with LLD meeting DSM-IV criteria (60+ years, 8 M, 21 F) and 29 matched controls with no history of depression (7 M, 22 F). MADRS, activities of daily living, and cognition scales were completed.	To test the utility of a novel wrist-worn device combined with deep learning algorithms to detect speech as an objective indicator of social interaction in LLD and in controls.	Algorithms were developed to classify: 1. speech and non-speech, and 2. wearer speech from other speech using audio recordings captured by the wearable device.	Participants with LLD produced less speech and reported poorer social and general functioning. Total speech activity and proportion of speech produced were correlated with attention and psychomotor speed but not depression severity or social functioning.
Lee et al. (56)^#^.	61 individuals (60+ years, 18 M, 43 F) with major depressive disorder according to DSM-IV-TR criteria and 143 age-matched healthy controls (50 M, 93 F).	To develop a voice-based screening test for depression measuring vocal acoustic features of elderly Korean participants.	Participants read mood-inducing sentences. Variations in 2,330 acoustic speech features derived from AVEC 2013 (e.g., loudness, MFCCs, zero crossing rate) and eGeMAPS (e.g., F0, jitter, shimmer, and HNR) were assessed.	Spectral and energy-related features could discriminate men with depression with 86% accuracy. Prosody-related features could discriminate women with depression with 77% accuracy.
Albuquerque et al. (57).	112 individuals (35–97 years, 56 M, 56 F). Anxiety and depression symptoms were assessed using the Hospital Anxiety Depression Scale.	To determine if variations in acoustic measures of voice are associated with non-severe anxiety or depression symptoms in adults across lifetime.	Reading vowels in disyllabic words and the “Cookie Theft” picture description task. 18 acoustic features extracted (e.g., F0, HNR, speech and pause duration measures).	Increased depression symptoms were associated with longer total pause duration and shorter total speech duration. Older participants tended to have more depressive symptoms.

*M, male; F, female; DSM, Diagnostic and Statistical Manual of Mental Disorders; PHQ-9, Patient Health Questionnaire-9; MADRS, Montgomery-Asberg Depression Rating Scale; LLD, late-life depression; CES-D, Center for Epidemiological Study Depression Scale; MFCCs, Mel Frequency Cepstral Coefficients; LPCCs, linear predictive coding coefficients; F0, fundamental frequency, HNR, harmonics-to-noise ratio*.

# *This study specifically assessed speech differences between males and females (56)*.

The use of NLP in LLD presents unique challenges with regard to understanding the specificity of identified speech alterations. Older adults are more likely to have additional medical comorbidities that may also cause speech changes (59, 60). For example, depression is both an independent risk factor and a prodrome for dementia (61), which makes an underlying neurocognitive disorder a potential confounding factor in detecting speech pattern changes (62). Second, older adults are more likely to be on multiple prescribed medications, which introduces the confounding factor of medication effects on the acoustic properties of speech (63). Compared to younger adults, older adults have been found to have a lower HNR, a marker of turbulent airflow generated at the glottis during phonation (64, 65), which may be partly attributable to the effects of medications (e.g., vocal tract dryness and thickened mucosal secretions). Finally, normal age-related hormonal and structural changes (e.g., cartilage ossification, muscle degeneration) (64, 65), may also contribute to lower HNR during phonation. These factors highlight the importance of including tightly matched control groups and large sample sizes to broadly examine how different factors impact speech measures in the older adult population.

Despite these challenges, the use of NLP approaches in older adults provides opportunities to improve our understanding of depression in the context of biological sex, medical comorbidities, and other clinical factors. For example, speech changes in mild cognitive impairment (MCI) and Alzheimer's disease (AD) have been well documented in recent years (66–68), and share commonalities with speech changes related to depression. Increased pause duration, increased pronoun use and reduced lexical and syntactic complexity have all been reported as occurring in MCI and AD (66, 67, 69, 70). Due to these similar changes in speech patterns, care must be taken to differentiate dementia from depression when using speech analysis tools, to avoid misdiagnosis. One study compared LLD to those with early AD on a picture description task, and found that those with AD had reduced informativeness of their descriptions, suggesting that measures of content may be useful in differentiating depression from AD (51). Two recent studies suggested that certain acoustic speech features may help differentiate depression and dementia, or dementia with and without comorbid depression (66, 71), but this topic requires further research.

Studies comparing the speech rate of LLD vs. Parkinson's disease (PD) consistently show that rate of speech is significantly reduced in LLD, whereas the finding is not consistent in PD. Individuals with PD may exhibit decreased, increased, or typical speech rates (41, 72). These findings reflect underlying pathophysiological changes in PD such as compensation for hypokinetic muscle tone, which is not seen in LLD. These speech differences may serve as useful markers to detect or monitor for depression in PD given the high degree of comorbidity between these two diagnoses.

Finally, applying NLP using a disease-agnostic or transdiagnostic approach may also play an important role in addressing the comorbidity seen in LLD. For example, apathy is a transdiagnostic symptom seen in MDD, schizophrenia, traumatic brain injuries, AD, PD, and other neuropsychiatric disorders. A study by Konig and colleagues found that the presence of apathy was associated with shorter speech, slower speech, and lower variance of prosody (lower F0 range) (73). Thus, transdiagnostic markers like apathy may be a helpful method of discriminating which speech features are unique to a disorder vs. those that may be shared across diseases and disorders.

## Complementary and Novel Strategies to Measure Depression

While current NLP approaches to capture symptoms of LLD are promising, there remain new opportunities to improve our understanding and implementation of this important research area. Over the past decade, advances in computer and mobile phone technologies have improved the quality and quantity of audiovisual input and output, allowing internet-based video clinical assessments to become more commonplace (74). The COVID-19 pandemic in particular has further led to a dramatic shift to online virtual care (75). With these shifts, NLP can potentially be applied to speech signals in real-time or asynchronously in clinical contexts. Recent studies have used NLP to generate COVID-19 phenotypes (76), track emotional distress in online cancer support groups (77), diagnose PD (78, 79), predict driving risk in older adults (80), and predict binge-eating behaviors (81).

These advances offer the potential of solving many of the challenges described in previous sections. For example, speech could be unobtrusively measured during routine visits on virtual care platforms between a healthcare provider and a patient. Data from these visits could be captured longitudinally and monitored for signs of depression, cognitive changes, or comorbid conditions. Individuals with depression in remission could conversely be monitored for signs of relapse. Preliminary studies have shown that integration of real-time audiovisual analysis into telemedicine platforms may be a feasible method of detecting an individual's emotional state (82). Additionally, the use of smartphone and wearables technology to record these features have also demonstrated feasibility and acceptability in initial pilot studies (55, 83).

Beyond the clinician-patient interaction, NLP could also be implemented in non-clinical settings. Recent advances in smart speaker technology (e.g., Amazon Alexa, Google Assistant, Microsoft Cortana, Apple Siri) has resulted in significant consumer adoption, and these devices are currently being investigated as tools to support independent living and wellness in older adults (84). These technologies may provide an opportunity for naturalistic speech to be collected longitudinally over time and may be a more granular and accurate method of detecting speech changes seen in depression (37). Advantages of smart speaker technologies include increased ease of use compared to computers and smartphones. Overall, these devices may hold promise to help older adults maintain independence through the use of passive monitoring for both depression and mild cognitive impairment and could serve as an “early warning system” to alert caregivers or professionals to negative symptom changes (84–86). However, the translation of AI technologies for home use in elderly populations may be limited without explicitly addressing ethical and legal considerations for patients, caregivers, and healthcare providers (87).

Regardless of the technology implemented, it is important to recognize that most of these technologies have not been specifically designed with older adults in mind. Older adults have reported hesitation about using novel technologies due to limited experience, frustration with technology, and physical health limitations (e.g., visual impairment) (88). From a privacy perspective, concerns have been raised by participants who may be unsure about how their electronic health data may be used, processed, or stored (89). Preliminary studies suggest these concerns can be mitigated with detailed informed consent from participants and by outlining privacy protocols in place (55). Ensuring that these technologies are culturally-adapted is another important consideration that can affect use and adoption (90). Finally, older adults have been shown to respond better to digital assistants with a socially-oriented interaction style (e.g., embedding informal conversation, using small talk, and encouragement) rather than assistants with a task-oriented style (e.g., structured formal responses) (91). As a result, there has been greater focus on embedding these interactions into automated social chatbots and companion robots for older adults (92). Tailoring these technologies to older adults has the potential to reduce technology hesitancy, improve adoption, and potentially increase the reliability of data that is collected as well (93).

## Conclusion

With an aging population globally, the identification and treatment of depression in older adults is critical. NLP approaches are proving to be a promising means to help assess, monitor, and detect depression and other comorbidities in older individuals based on speech. However, additional research is needed to fully characterize the spectrum of depression symptoms experienced by older individuals. Complementary speech collection and analysis strategies using AI, wearables, and other novel technologies may help further advance this important field.

## Author Contributions

DD and AY contributed to the conception, design of the study, and wrote the first draft of the manuscript. JR and MG wrote sections of the manuscript. All authors contributed to manuscript revision, read, and approved the submittedversion.

## Conflict of Interest

DD, JR, and MG are employees of Winterlight Labs. AY is a medical consultant for Winterlight Labs.

## Publisher's Note

All claims expressed in this article are solely those of the authors and do not necessarily represent those of their affiliated organizations, or those of the publisher, the editors and the reviewers. Any product that may be evaluated in this article, or claim that may be made by its manufacturer, is not guaranteed or endorsed by the publisher.
